# Mutations in *TULP1*, *NR2E3*, and *MFRP* genes in Indian families with autosomal recessive retinitis pigmentosa

**Published:** 2012-05-04

**Authors:** Chitra Kannabiran, Hardeep Singh, Nishika Sahini, Subhadra Jalali, Gayathri Mohan

**Affiliations:** 1 Kallam Anji Reddy Molecular Genetics Laboratory, Hyderabad Eye Research Foundation, Andhra Pradesh, India; 2 Smt. Kannuri Santhamma Centre for Vitreoretinal diseases, Andhra Pradesh, India; 3 L.V. Prasad Eye Institute, Hyderabad, Andhra Pradesh, India

## Abstract

**Purpose:**

To identify genes underlying autosomal recessive retinitis pigmentosa (ARRP) by homozygosity mapping.

**Methods:**

Families with ARRP were recruited after complete ophthalmic evaluation of all members and diagnosis of RP by predefined criteria. Genomic DNA from affected members of 26 families was genotyped on Illumina single nucleotide polymorphism (SNP) 6.0 K arrays with standard procedures. Genotypes were evaluated for homozygous regions that were common and concordant between affected members of each family. The genes mapping to homozygous intervals within these families were screened for pathogenic changes with PCR amplification and sequencing of coding regions. Cosegegration of sequence changes with disease was determined within each pedigree, and each variation was tested for presence in 100 unrelated normal controls.

**Results:**

A genome-wide scan for homozygosity showed homozygous regions harboring the tubby like protein 1 gene (*TULP1*; chromosome 6) in one family, the nuclear receptor subfamily 2, group E, member 3 gene (*NR2E3*; chromosome 15) in three families, and the membrane frizzled-related protein gene (*MFRP*; chromosome 11) in one family. Screening of the three genes in the respective families revealed homozygous disease-causing mutations in three families. These included a missense mutation in *TULP1*, a deletion-cum-insertion in *NR2E3*, and a single base deletion in *MFRP*. Patients from all three families had a rod-cone type of dystrophy with night blindness initially. The *NR2E3* and *MFRP* genes were associated with fundus features atypical of RP.

**Conclusions:**

This study shows involvement of the *TULP1*, *NR2E3*, and *MFRP* genes in ARRP in Indian cases. Genome-wide screening with SNP arrays followed by a prioritized candidate gene evaluation is useful in identifying genes in these patients.

## Introduction

Retinitis pigmentosa (RP) is a group of hereditary retinal degenerations involving loss of retinal photoreceptor cells and leading to irreversible visual loss or blindness. The age of onset of RP is usually within the first or second decade, and it is a progressive disorder with a high degree of clinical heterogeneity. Though a range of different yet overlapping phenotypes comprise RP, the characteristic features are initial symptoms of diminished night and/or day vision, progressive loss of visual acuity and visual fields, extinguished electroretinographic (ERG) responses, and fundus changes, including arterial attenuation, disc pallor, retinal pigment epithelium (RPE) atrophy, and the presence of intraretinal pigmentary deposits. Mutations in more than 60 genes are known for autosomal and X-linked forms of non-syndromic RP alone (RetNet). Autosomal recessive RP (ARRP) accounts for one-third or more of the total cases of RP in different populations [1] and has the highest degree of genetic heterogeneity among the various forms, with 34 genes reported so far.

Autozygosity or homozygosity screening to identify loci for recessive disorders has been recognized as a powerful and effective approach especially in the presence of inbreeding and consanguinity [2]. Application of this method on a genome-wide scale has been facilitated by the use of SNP arrays to detect regions of homozygosity, with subsequent candidate gene analysis [3]. The main advantages of this approach are that it can be applied in small families with even a single to a few affected individuals that would not be significant enough for linkage analysis and, in the case of a highly heterogeneous disorder such as RP, can enable the search for disease genes to be narrowed to a few or more potential loci that can be evaluated individually for known and novel candidates. The prevalence of RP varies in different parts of the world, ranging from about 1 in 4,000 to 1 in 1,000 [4-6]. Blindness due to RP is estimated to occur in 1 in 1,000 of the population of Andhra Pradesh in south India [7]. Knowledge of causative genes in each population can facilitate the design of screening and counseling strategies for patients, and serve to direct or develop appropriate therapeutic approaches that are genotype-dependent. There is relatively little knowledge of genes underlying RP in Indian populations. We performed a genome-wide homozygosity screening on 6.0K SNP arrays, and herein we report novel mutations in known RP genes detected in three families with autosomal recessive RP (ARRP).

## Methods

### Patient recruitment

Families were recruited based on ophthalmic evaluation of probands and all available family members, including parents and siblings. All subjects underwent a comprehensive examination, including visual acuity testing, indirect ophthalmoscopy, ERGs, and visual fields. Diagnosis of RP was made according to predefined criteria, which have been previously described [8]. Pedigree information including the presence and nature of consanguinity was obtained in each family. Those with a pattern suggestive of autosomal recessive inheritance were included. The protocol for the study was approved by the Institutional Review Board. Informed consent was obtained from all subjects for participation in the study and collection of blood samples. Peripheral blood was collected by venipuncture in EDTA-coated vaccutainer tubes and stored at −20 °C until extraction of DNA.

### Single nucleotide polymorphism arrays and genotyping

Genomic DNA was isolated from blood leukocytes with standard methods. DNA was quantified using the PicoGreen assay (Invitrogen Life Technologies, Carlsbad, CA). DNA from two affected members from each family was genotyped on the Illumina SNP 6.0K genotyping array (Illumina Inc., San Diego, CA). Hybridization to the array was performed according to the Infinium II assay protocol using reagents from Illumina. Briefly, genomic DNA was denatured, and subjected to whole genome amplification. Amplified DNA was fragmented and hybridized on the Infinium Array for 24 h under appropriate conditions. Excess unhybridized DNA was washed, and single base extension was performed on hybridized primers with labeled nucleotides. Arrays were stained, washed, and scanned on the Illumina Bead Array Reader. SNP genotypes were obtained using the Illumina Bead Studio v 2.0 genotyping module. The genotypes call rates obtained were around 98%. SNP data were arranged according to genomic position and copied into an Excel (Microsoft Corporation, Redmond, WA) spreadsheet, and runs of autozygosity were detected using an Excel-based program created for this purpose. Regions of homozygous SNPs with at least ten or more consecutive SNPs were selected for further evaluation. Consecutive and homozygous SNPs common to two affected siblings within each family were selected, and the ten largest such regions were shortlisted to identify candidate genes. In the initial screening phrase, regions that were 5 Mb or more were screened first. Regions with one or more SNP “no calls” in the absence of at least ten consecutive homozygous SNPs were not considered, since the presence of heterozygous genotypes for the failed SNPs cannot be excluded. In such cases, if the genotypes within the corresponding region were confirmed in one of the affected siblings in a family, that genomic region was considered a candidate. Selected homozygous regions were reconfirmed by genotyping microsatellite markers within the interval in the respective families. These regions were evaluated on the ABI3130 XL platform using GeneScan software (Applied Biosystems Inc., Palo Alto, CA).

### Candidate gene screening

Known retinal disease genes localizing to autozygous genomic regions of ARRP-affected individuals were screened for pathogenic changes. Coding regions were PCR-amplified and directly sequenced to identify any changes. Pathogenicity of sequence changes was further confirmed with cosegregation analysis in family members and in 100 unrelated normal controls of Indian origin. Controls were recruited locally and were mostly southern Indian in origin. Missense changes were evaluated with multiple sequence alignments using NCBI BLAST (Basic Local Alignment Search Tool), SIFT (Sorting Intolerant From Tolerant) [9], and Polymorphism Phenotyping (PolyPhen) [10] to predict the impact on the protein.

## Results

Twenty-six families were included in the study, of which 23 were consanguineous. All families were from southern India. Following genome-wide SNP genotyping of affected members of 26 families, homozygous segments of ten or more consecutive SNPs shared between affected individuals of each family were shortlisted for candidate gene evaluation. There were several such homozygous segments in each family, ranging from one to 18 in the families studied (Table 1), with an average of about nine homozygous segments per family. The sizes in megabases of the regions defined by >ten consecutive SNPs ranged from 1 to 45 Mb. Affected members of 16 out of 26 families were found to have homozygous regions in their genome harboring one or more of 18 different genes for recessive RP. These genes were evaluated for possible pathogenic changes in relevant families. Pathogenic mutations were identified in three different genes in three families. The details of the remaining 23 families, including the number of autozygous regions found in each family and the known genes if any tested in each family, are summarized in Table 1.

**Table 1:** Details of ARRP families screened

**Family number**	**No. of homozygous regions of >10 consecutive SNPs**	**RP genes tested for mutations**	**Size of corresponding homozygous region (Mb)**	**HBD confirmed by microsatellite markers**
1	8	*CRB1*	21.2	Y
		*AIPL1, GUCY2D*	4.3	N
2	11	*-*	-	-
3	11	*RP1*	8.8 Mb	Y
4	12	*-*	-	-
5	6	*NR2E3*	9.6	Y
6	7	*-*	-	-
7	5	*-*	-	-
8	13	*RGR*	40.9	Y
9	15	*CRB1*	15.3	Y
10	7	*-*	-	-
11	18	*-*	-	-
12	10	*-*	-	-
13	7	*RDH12*	44.1	Y
14	11	*RGR, PCDH21*	23.6	Y
		*FAM161A*	8.2	N
15	12	*-*	-	-
16	6	*-*	-	-
17	8	*-*	-	-
18	15	*FAM161A*	5.7	N
19	5	*RBP3*	26	Y
20	6	*RDH12*	23.3	Y
		*RD3*	18.6	N
21	13	*NRL*	9.5	N
22	8	*NR2E3, RLBP1*	28.8	Y
		*FAM161A*	15.2	Y
23	6	*RDH5*	7.5	Y

Shown above are the 23 families screened for homozygosity on the SNP 6.0 K array, with mutations not identified. The numbers of homozygous intervals in each family, consisting of more than 10 consecutive SNPs each, are shown in column 2. Known RP/retinal dystrophy genes localizing to one or more homozygous regions of 13 families were screened for mutations (shown in column 3). Genes mapping to a common region are shown in the same row. The size of the homozygous segment corresponding to each of the genes is shown in column 4. The last column shows whether the homozygosity at the relevant locus was confirmed by testing affected offspring and at least one unaffected sibling/parent by using microsatellite markers. HBD- homozygosity by descent.

The *TULP1* and *MFRP* genes mapped to the homozygous regions in one family each, and the *NR2E3* gene mapped to homozygous regions in three out of 26 families. Screening of these genes revealed novel pathogenic mutations in the three families, one each with mutations in *TULP1*, *NR2E3*, and *MFRP*. Each mutation was confirmed for cosegregation in family members (parents and siblings) as well as for absence in 100 unrelated normal controls. The mutations described here were absent in the control population.

### *TULP1* gene

**Figure 1:**
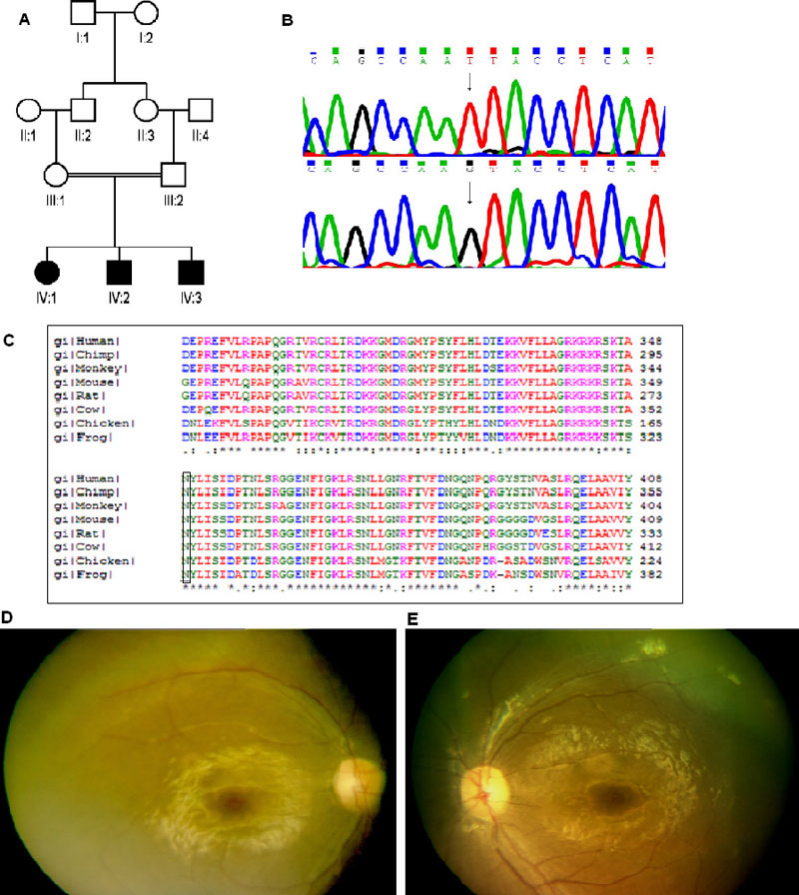
Details of ARRP patient from Family A with a mutation in the *TULP1* gene. **A**: Pedigree shows affected (dark symbols) and unaffected (open) symbols, with squares representing men and circles representing women. A double line connecting spouses denotes consanguinity. **B**: Sequence chromatogram of the *TULP1* gene in normal control (top) and in patient A-1 (bottom) with homozygous mutation T>G (arrows), resulting in a codon change AAT (*Asn*) to AAG (*Lys*). The protein sequence alignment of TULP1 from different species (**C**) shows conservation of the Asn349 residue. Fundus photograph of right (**D**) and left (**E**) eyes of patient A-1 taken at age nine years shows greyish discoloration of the retina due to widespread RPE atrophy, severe arterial narrowing, disc pallor, and cellophane retinopathy due to a thin epiretinal membrane.

The *TULP1* gene was screened in Family A (Figure 1A) due to the presence of homozygosity at this locus extending to 8.1 Mb. A homozygous missense change c.1047T>G (NM_003322.3) was detected in two affected individuals, corresponding to Asn349Lys (Figure 1B). A blood sample was not collected from one of the affected individuals as the patient was mentally challenged. The unaffected parents were carriers of the mutation. This mutation was not found in 100 unrelated normal controls. The Asn349 residue is located in the conserved C-terminal tubby domain. Multiple sequence alignment of the protein shows that this residue is highly conserved (Figure 1C). Analysis of the substitution with the SIFT and PolyPhen tools predicted that the change is probably damaging to the protein.

### Clinical features

The affected members in this family had a history of onset of poor vision with night vision worse than daytime vision before ten years of age (Table 2). Two of the affected children reportedly had visual defects before the age of one year according to the parents. The patients complained of difficulties in day and night vision. They had slowly progressive vision loss. Fundus evaluation showed RPE degeneration, arterial narrowing, pigmentary deposits, and disc pallor. In addition, cellophane reflexes from the macula were observed. There was diffuse loss of visual fields. Scotopic and photopic flash ERG was extinguished in one patient, and visual acuity was about 20/100. In the other patient, ERG was not performed. Clinically, the patients had typical RP with the onset at an early age and progressive disease. The fundus photographs of one patient in this family are shown in Figures 1D,E.

**Table 2:** Clinical features of patients with ARRP

**Patient id**	**Gene involved**	**Age at onset**	**Age at presentation/sex**	**Symptoms**	**Visual acuity**	**Fundus features**	**ERG**	**Fields**
A-1	TULP1	8 year	14 years /M	Decreased vision (night> day) & decreased night mobility	20/100 OU	Arterial narrowing; disc pallor, RPE degeneration; cellophane reflexes	Not done	Diffuse loss, not reliable
A-2	TULP1	1 year	9 years/M	20/40 OD; 20/30 OS			Extinguished	Diffuse loss,
B-1	NR2E3	4 year	10 years/M	Decreased night vision	20/20 OD; 20/25 OS	White flecks mid-periphery; healed gliotic scars at posterior pole	Sub-normal rod-cone type	Peripheral field loss
B-2	NR2E3	4 year	7 years/M	Decreased night vision; squint left eye	20/25 OD; 20/400 OS (strabismic amblyopia)			
C-1	MFRP	10 year	29 years/M	Decreased night vision	20/200 OD; 20/400 OS	Diffuse marbelised RPE degeneration, few pigments in periphery and minimal disc pallor	Extinguished	10 degrees
C-2	MFRP	10 year	28 years/M	Blurred vision, progressive nyctalopia	20/100 OD 20/100 OS Hyperopia +13D		Sub normal rod cone type, scotopic and photopic flash ERG	Diffuse periphery, field loss 20 degrees
C-3	MFRP	10 year	21 years/M	Decreased night vision	20/60 OD; 20/50 OS. Hyperopia +11D		Sub normal rod cone	10 degrees

The table shows a summary of clinical features of affected patients that were clinically evaluated from each of the 3 ARRP families with mutations identified.

### *NR2E3* gene

**Figure 2:**
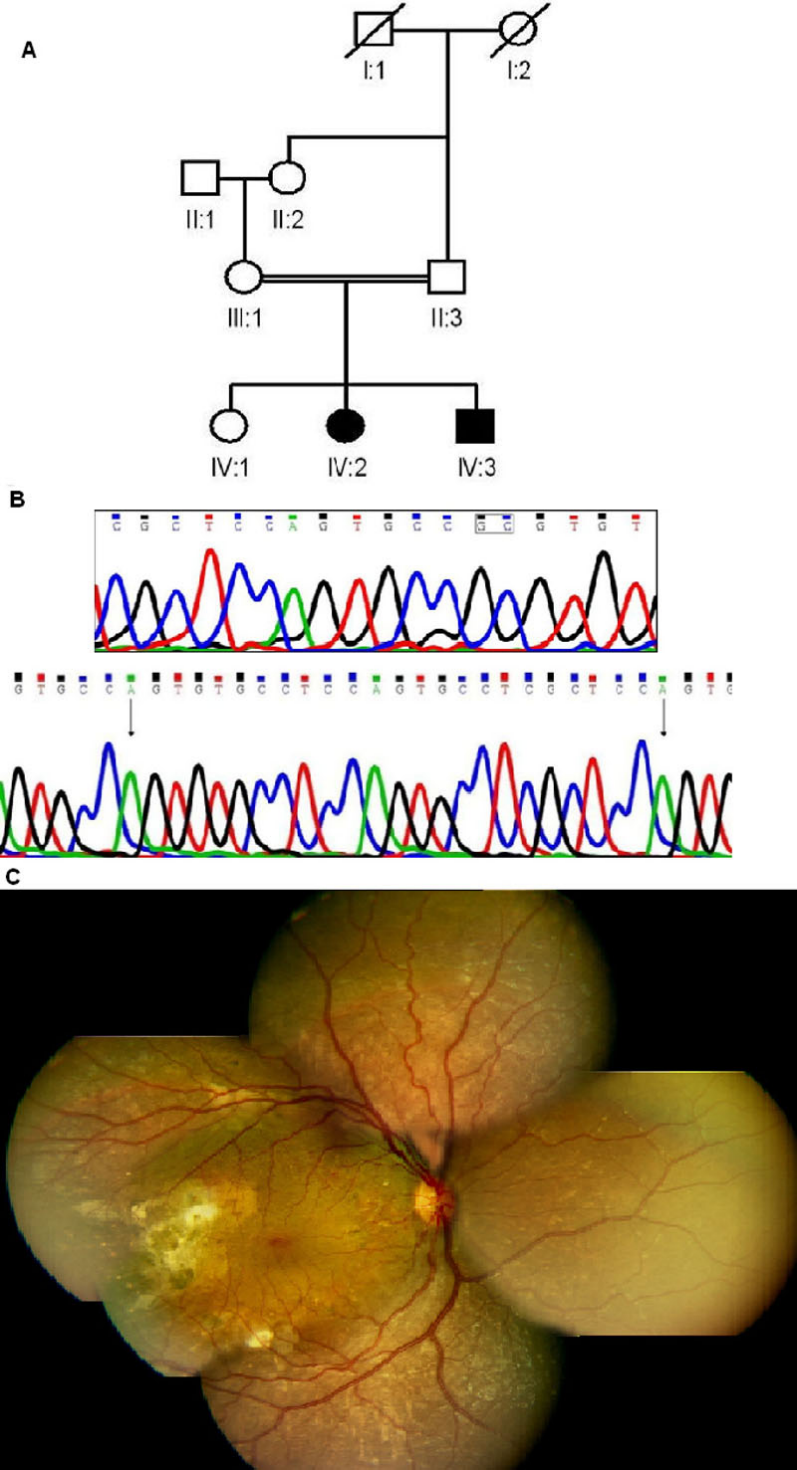
Molecular and clinical details of a patient from Family B with a mutation in the *NR2E3* gene. **A**: Pedigree is shown (explanation of symbols as in Figure 1). **B**: Sequence of the *NR2E3* gene in normal control (top) and patient B2 with homozygous deletion+insertion (bottom). The dinucleotide undergoing deletion is boxed in top panel. The arrows in the bottom panel mark the inserted sequence. **C**: Fundus montage of right eye of patient B-2 (aged 10 years) with *NR2E3* mutation showing peripheral graying of retina with white flecks due to RPE atrophy with macular sparing with hardly any disc or arterial changes. The right temporal retina had unexplained sub-retinal scarring/gliosis and no obvious bone corpuscular pigment migration at this age.

Affected members of three families had homozygous segments mapping to the chromosome 15 region of 41 Mb, 28 Mb, and 9.5 Mb. The largest homozygous segment of 41 Mb included the *NR2E3* and *RLBP1* genes present in Family B (Figure 2A). Screening of the coding regions of *NR2E3* and *RLBP1* genes in the respective families showed a homozygous complex mutation in Family B that consisted of a deletion of GC at codon 48 (exon 2) with the insertion of 25 bp (c.143–144delGCins25; NM_014249.2; Figure 2B). The mutation predicts a frameshift at codon 48 with 65 amino acids of the altered reading frame followed by a premature termination codon. This mutation was present in both affected siblings and was heterozygous in both parents. The remaining unaffected sibling was not available for the study. This mutation was absent in 100 unrelated normal controls. No mutations in *NR2E3* or *RLBP1* were found in the remaining two families.

### Clinical features

The two affected individuals in Family B had initial symptoms of decreased night vision with onset of disease in the first few years of life (Table 2). The fundus showed features of an atypical retinal dystrophy with white flecks in the periphery and gliotic scars in the posterior pole (Figure 2C). ERGs were of the rod-cone type. The condition of the ERG and clinical features were highly suggestive of an enhanced S-cone syndrome (ESCS) phenotype [11]. However, ESCS could not be confirmed as the patient did not undergo optical coherence tomography for macula schisis. In addition, the gliotic scars observed were unusual.

### *MFRP* gene

**Figure 3:**
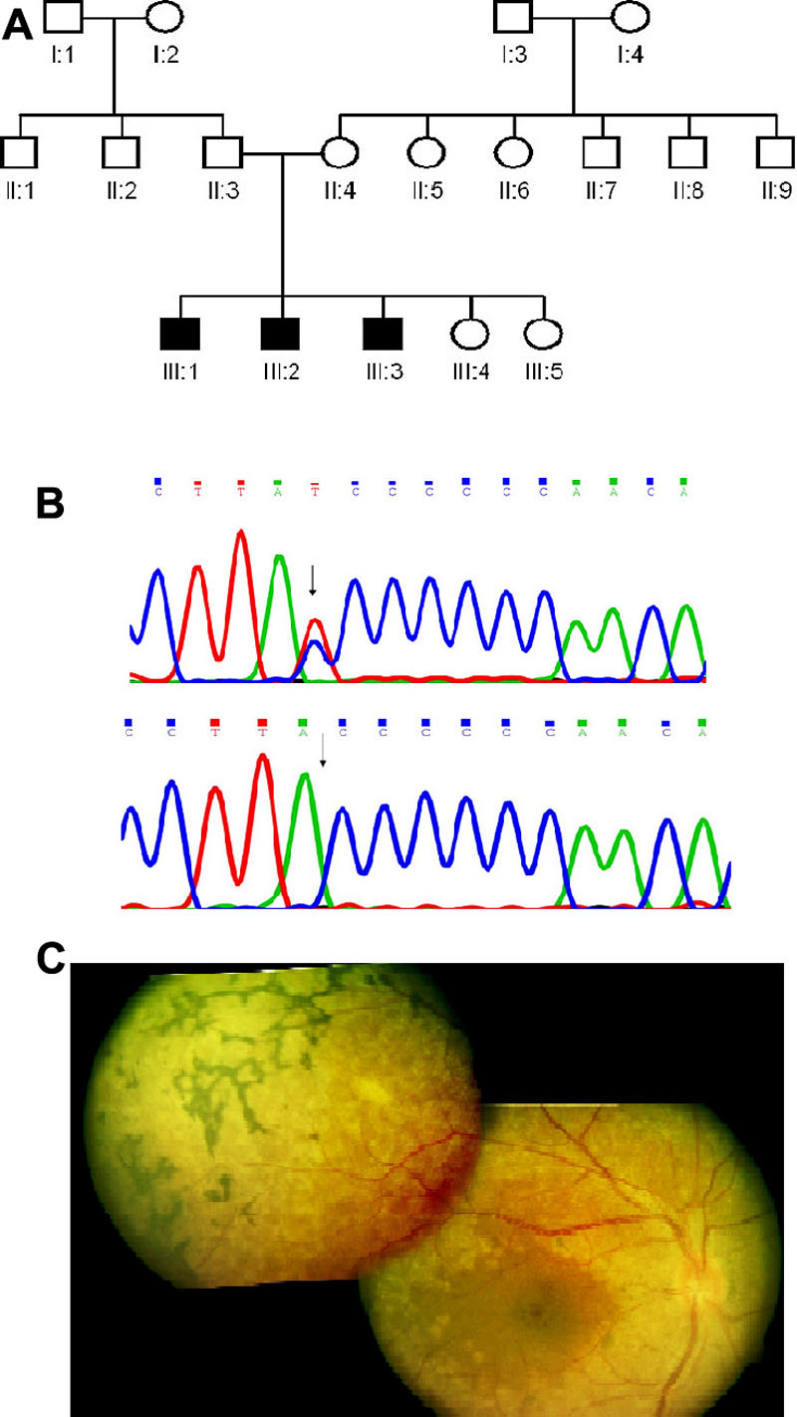
Molecular and clinical details of patient from Family C with a mutation in the *MFRP* gene. **A**: The family pedigree is shown. **B**: Sequence of *MFRP* gene in normal control (top panel) and in patient C-1 (bottom panel). The arrows in the top and bottom panels respectively, mark the SNP c.492C>T (rs36015759) and the position of the single base deletion in patient C-1. **C**: Fundus montage of the right eye of patient C-1 (aged 21 years) from Family C with an *MFRP* gene mutation showing perifoveal pigment deposits, relative parafoveal sparing, diffuse extensive graying of retina with white flecks extending from arcades to the peripheral retina, and the presence of a peripheral reticular, bone corpuscular type of pigmentary retinopathy. There is not much disc pallor or arterial narrowing.

This gene was screened in Family C (Figure 3A) due to the presence of an 8.5 Mb homozygous region on chromosome 11 that included the *MFRP* gene locus. All three affected had a single base deletion in exon 5, c.498delC, at codon 166 of the protein (Pro166ProfsX26; NM_031433.2; Figure 3B). The parents were heterozygous carriers. Screening of 100 unrelated normal controls showed this change was absent in the control population. The frameshift resulting from this deletion leads to termination after 25 residues.

### Clinical features

The three affected individuals showed the onset of symptoms very early in life noted from childhood at about ten years or younger, based on the history of visual defects reported by the patients. The individuals had progressive diminution of night vision. The fundus showed some degree of arterial narrowing, disc pallor, and confluent RPE degeneration including perifoveal pigments (Table 2; Figure 3C). The bright flash ERGs were sub-normal and severely diminished with a rod-cone pattern. Clinically, all the patients had atypical RP wherein the younger two affected siblings (C2 and C3 in Table 2) had fewer pigments with high hyperopia of +11 and +13 diopters, respectively. The oldest sibling (C1) had slightly more pigments and macular involvement than the others.

## Discussion

The results described in this study identify mutations in ARRP genes in families of Indian origin. These data add to and expand on our previous studies on ARRP [8,12]. Identifying potential disease loci by homozygosity mapping particularly for a heterogeneous disorder is facilitated by SNP arrays due to the rapidity with which whole genome analysis is possible. This approach has resulted in known and novel genes for ARRP being detected in previous studies [13-15]. We found 16 families had loci mapping known ARRP or related retinal dystrophy genes, and screening of these revealed mutations in three families. This is approximately one-fifth of the families screened for mutations in known genes. The disease locus could map to any of the other homozygous regions mapped in the rest of the cases. Since the families involved are not suitable for linkage analysis, one cannot assess the significance of the homozygous regions detected here. Rather, this approach provides plausible candidates for testing and may help narrow the potential loci to be screened in a subset of families. In the presence of a high degree of consanguinity, one would also expect a higher prevalence of homozygosity in the genome in such families [3].

The *TULP1* mutation of Asn349Lys is located in the conserved tub domain extending between residues 298 and 536 of TULP1 (AAB53700.1) and is associated with a phenotype of early-onset retinal dystrophy with onset at one and eight years for two patients in the family (Table 2). Notable features included nyctalopia in early stages, early macular involvement in one of the siblings, and bright macular reflexes in both cases. The presence of macular involvement was also a feature of patients with a mutation in *TULP1* reported in our earlier study [12]. Contrary to reports in the literature in which nystagmus was a feature of patients with *TULP1* mutations [3,15,16], no nystagmus was present in our patients. Other clinical features were characteristic of RP.

The complex novel mutation in *NR2E3* detected in our study predicts a frameshift in the N-terminal region of the protein and hence would be expected to result in complete loss of activity. Several mutations have been reported in *NR2E3* with the majority being missense mutations and only a few deletions or frameshifts [17]. Mutations in *NR2E3* have been associated with a range of overlapping phenotypes, including ESCS, clumped pigmentary retinal degeneration, Goldman-Favre syndrome, and dominant as well as recessive RP. Considerable phenotypic variability is a feature of *NR2E3* mutations although features of the *NR2E3*-linked recessive disorders include night blindness in early stages, clumped pigmentary deposits, and a more severe loss of rods than cones [17]. In addition, the same mutation is reported to be involved in different phenotypes such as ESCS and ARRP [18,19]. Patients in the present study lacked the more typical features of RP but had a rod-cone phenotype without the characteristic clumped pigmentary deposits. The ERGs were of a subnormal rod-cone type. In addition, notable features seen in our cases were white flecks in the mid-periphery and sub-retinal scars. In our patients, the phenotype and ERG were also descriptive of ESCS. However, the patient did not undergo optical coherence tomography, and we could not confirm the clinical description of ESCS.

The single base deletion in the *MFRP* gene observed in the present study is interesting in that it represents the fifth independent occurrence of the same mutation reported, in patients of different countries of origin. The C residue at position 498 is part of a stretch of seven C residues from c.492 onwards that represents a mutational hotspot for this gene, since the mutation at this site has occurred in patients of different populations, including Mexican [20,21], Spanish [22], and Caucasian [23], and patients from the UK [24]. However, different studies have designated the mutations at this site differently at DNA as well as protein levels; the c.492delC [23]and c.498delC [21,22] changes as reported are not really distinguishable from each other based on the DNA sequence since it is not clear which C residue among the seven consecutive ones is undergoing deletion. According to recent nomenclature guidelines (mutnomen), in case of ambiguity, the 3′ most position is assigned to have the mutation; hence, we have described it as c.498delC. Interestingly, one of the mutations at this site is an insertion of C [20]. Nine distinct mutations have been reported so far in *MFRP* associated with variable phenotypes. In Sundin et al.’s study [23], the phenotype consisted of nanophthalmos, and no retinal degeneration was observed. In subsequent studies of patients with *MFRP* mutations, consistent features frequently reported are the early onset of visual loss [24], the presence of high hyperopia, diminished ERG responses with a rod-cone pattern, and RPE atrophy [20,21]; occasional or inconsistent features include night blindness, hyperpigmentation of the retina, foveoschisis, macular cysts, and optic nerve drusen [22,24]. In the present study, features of RP were seen without the other features of *MFRP*-related oculopathy. Affected siblings in the present study had night blindness, rod-cone type of ERG, and extensive degeneration of RPE. High hyperopia was observed in this family though variable; the presence of macular involvement was also variable (Table 2).

In summary, the data presented here demonstrate the role of the *TULP1*, *MFRP*, and *NR2E3* genes causing ARRP in Indian families, report novel mutations in these genes, and add to the range of mutations and clinical phenotypes that have been documented for this heterogeneous group of disorders.
